# Synthesizing Electrodes Into Electrochemical Sensor Systems

**DOI:** 10.3389/fchem.2021.641674

**Published:** 2021-03-31

**Authors:** Yulia G. Mourzina, Yuri E. Ermolenko, Andreas Offenhäusser

**Affiliations:** ^1^ Institute of Biological Information Processing - Bioelectronics (IBI-3), Forschungszentrum Jülich, Jülich, Germany; ^2^ Institute of Chemistry, Saint Petersburg State University, Saint Petersburg, Russia

**Keywords:** nanowire assembly, hydrogen peroxide, manganese porphyrin, plant analysis, biomimetic sensor material, electrochemically reduced graphene oxide

## Abstract

Electrochemical sensors that can determine single/multiple analytes remain a key challenge in miniaturized analytical systems and devices. In this study, we present *in situ* synthesis and modification of gold nanodendrite electrodes to create an electrochemical system for the analysis of hydrogen peroxide. The sensor system consisted of the reference and counter electrodes as well as the working electrode. Electrochemical reduction of graphene oxide, ErGO, on the thin-film gold and gold nanodendrite working electrodes was used to achieve an efficient sensor interface for the adsorption of a biomimetic electrocatalytic sensor material, Mn(III) *meso*-tetra(N-methyl-4-pyridyl) porphyrin complex, with as high as 10^–10^ mol cm^−2^ surface coverage. The sensor system demonstrated a detection limit of 0.3 µM H_2_O_2_ in the presence of oxygen. Electrochemical determination of hydrogen peroxide in plant material in the concentration range from 0.09 to 0.4 µmol (gFW)^−1^ using the electrochemical sensor system was shown as well as *in vivo* real-time monitoring of the hydrogen peroxide dynamics as a sign of abiotic stress (intense sunlight). Results of the electrochemical determination were in good agreement with the results of biochemical analysis with the spectrophotometric detection. We anticipate that this method can be extended for the synthesis and integration of multisensor arrays in analytical microsystems and devices for the quantification and real-time *in vivo* monitoring of other analytes and biomarkers.

## Introduction

Hydrogen peroxide plays an essential role in developmental and adaptive responses in animal and plant cells (Neill et al., 2002; Quan et al., 2008; Kreslavski et al., 2012; Zandalinas and Mittler 2018; Smirnoff and Arnaud 2019). In plants, it is produced by electron transport in chloroplasts and mitochondria via superoxide, peroxisomal oxidases, plasma membrane NADPH oxidase, and other oxidases and peroxidases (Quan et al., 2008; Smirnoff and Arnaud 2019). As one of the reactive oxygen species, hydrogen peroxide has a dual function in cells. In addition to damaging cellular molecules and negatively affecting plant organisms, it can have functions in cell signaling and adaptation mechanisms (Neill et al., 2002; Kreslavski et al., 2012; Smirnoff and Arnaud 2019). During the last few years, there has been a particular focus on the role of H_2_O_2_ as a signaling molecule in plant responses to stress factors and pathogen defense, but the mechanisms are poorly understood. The development of methods for the quantification and real-time *in vivo* monitoring of hydrogen peroxide in plant tissue will enable a better understanding of its role in plant physiology, development, and adaptation.

Spectrophotometric, fluorometric, and chemiluminescent methods have been used to determine hydrogen peroxide in plant tissue (Patterson et al., 1984; Veljovic-Jovanovic et al., 2002; Cheeseman 2006; Zhou et al., 2006; Ortega-Villasante et al., 2016; Si et al., 2018; Smirnoff and Arnaud 2019). Recently, the detection of light-induced hydrogen peroxide production has been reported in PS II membranes using an electrochemical biosensor (Prasad et al., 2015). Bioelectronic applications of the plant tissue in electrochemical transistors have also been demonstrated opening up new perspectives for bioelectronic applications in plant science (Stavrinidou et al., 2015; Coppedè et al., 2017). Electrochemical sensors are highly promising for the quantification of hydrogen peroxide in plants, since they might offer quick analysis with a high spatio-temporal resolution due to miniaturization, the possibility for the *in vivo* monitoring of the plant physiological state, and they can be easily integrated into portable analytical microsystems.

There are three main directions for the development of electrochemical sensors to determine hydrogen peroxide.

Over the past few decades, there have been extensive developments to hydrogen peroxide biosensors based on bioelectrocatalysis by immobilized, natural and - more recently - genetically modified peroxidases (Lindgren et al., 2000; Ruzgas et al., 2002; Koposova et al., 2014; Koposova et al., 2015) as well as smaller heme-proteins such as cytochrome *c*, hemoglobin, myoglobin, and microperoxidase (Wollenberger et al., 2007). The studies are aimed at improving the direct electron transfer between catalytic redox centers of biomolecules and electrodes or "wiring" the enzyme to the electrode surfaces to eliminate the need for mediators in solutions (Vreeke et al., 1992; Schuhmann 2002; Prasad et al., 2015). Nanomaterials are used in this type of electrochemical biosensor to achieve and facilitate direct electron transfer. In addition, modification of electrodes with nanomaterials leads to an increase of the electroactive surface area and thus to a higher sensitivity per unit of the geometric surface. Furthermore, some studies report an improvement in the stability of the immobilized enzyme (Koposova et al., 2014; Koposova et al., 2015; Nikolaev et al., 2015).

A group of electrochemical sensors for hydrogen peroxide is based on the phenomenon of electrocatalysis on metal surfaces. The catalytic oxidation of hydrogen peroxide is due to the presence of a thin film of metal oxide on the electrode surface. Hydrogen peroxide reduces the surface oxide film to metal, which is followed by metal electrochemical reoxidation (Johnston et al., 1995; Gerlache et al., 1997; Katsounaros et al., 2012). However, a two-electron irreversible oxidation of hydrogen peroxide at anodic potentials, for example 0.6–0.7 V at a Pt electrode, is often affected by the interfering effects of substances oxidized at these potentials, such as ascorbic acid, acetaminophen, urates, dopamine, and other components of biological and natural media (Davis 2009). When registering the reduction of H_2_O_2_ at cathodic potentials, an interfering effect of oxygen reduction appears, since both substances are reduced at close potentials. Thus, unmodified metal electrodes have the disadvantage of poor selectivity in the presence of oxygen, which is present in approx. 0.27 mM concentrations in solutions under ambient conditions. The optimal electrode potential values are 0 V and small absolute values, where unwanted side processes of interfering substances do not contribute to the sensor response. Metal nanostructures are extensively investigated for the development of this group of electrochemical sensors. The modification of electrode surfaces with nanomaterials can be used to design metal surfaces with an optimized structure and energy for the electrocatalytic conversion of hydrogen peroxide (Guascito et al., 2008; Guascito et al., 2013; Chen et al., 2014; Huang et al., 2015; Chirizzi et al., 2016; Olenin 2017; Nikolaev et al., 2018a; Dhara and Mahapatra 2019; Huang et al., 2019; Peng et al., 2019).

Electrochemical sensors with sensor materials based on the principles of biomimetics have also motivated significant research activities. In this group of sensors, biomimetic compounds and nanozymes, mainly represented by the complexes and oxides of transition metals, are used to modify the electrodes (Morgan and Dolphin 1987; Batinić-Haberle et al., 2010; Wei and Wang 2013; Chen et al., 2014; Baglia et al., 2017; Jiang et al., 2018; Dhara and Mahapatra 2019; Huang et al., 2019; Wang et al., 2019; Wu et al., 2019; Wang et al., 2020). Nanomaterials are also used for the development of this group of sensors for a number of the above-mentioned reasons. Among the first sensors using the principles of biomimetic electrocatalysts were sensors based on the Fe(II)Fe(III) system, such as Fe_4_[Fe(CN)_6_]_3_ - Prussian Blue - “artificial peroxidase” (Karyakin 2001; Ricci and Palleschi 2005), and magnetite Fe_3_O_4_ (Wei and Wang 2013). The peroxidase-like properties of the Fe_3_O_4_ nanoparticles, “nanozymes,” were studied in 2007 (Gao et al., 2007) and later used to fabricate sensors based on the principles of biomimetics. These sensors were based on the electrocatalytic reduction of hydrogen peroxide and demonstrated detection limits in the range of μM concentrations (Lin and Leu 2005; Guivar et al., 2015). Studies are also being carried out on other transition metal complexes with biomimetic activity as well as metal-organic frameworks (Calas-Blanchard et al., 2014; Paolesse et al., 2017; Mourzina and Offenhäusser 2020; Peng et al., 2020; Wang et al., 2020). Recently, we studied a series of water-soluble Mn(III) porphyrin complexes for the electrocatalytic reduction of hydrogen peroxide in solutions (Mourzina and Offenhäusser 2020). An electrochemical sensor has been developed based on the Mn(III) *meso*-tetra(N-methyl-4-pyridyl) porphyrin complex, MnTMPyP, immobilized on the glassy carbon electrode, GCE, for the non-enzymatic selective electrocatalytic determination of hydrogen peroxide with a detection limit of 5 × 10^–7^ M (Peng et al., 2020). The use of the Mn porphyrin complex as a biomimetic sensor material made it possible to carry out determinations of hydrogen peroxide in the presence of oxygen. It was found that higher currents of the electrocatalytic reduction of hydrogen peroxide and sensitivity were observed in the presence of oxygen than in deoxygenated solutions in neutral, acidic, and alkaline media. Hence, the sensor works in the area of physiological pH values with high sensitivity and selectivity, and was successfully used for the analysis of biological media (Peng et al., 2020).

Here, we report on the *in situ* synthesis and modification of gold nanodendrite (AuND) electrodes prepared using the directed electrochemical nanowire assembly (DENA) to create an electrochemical system for the analysis of hydrogen peroxide. The electrochemical reduction of graphene oxide on the gold electrodes is used to create a sensor interface for the adsorption of the MnTMPyP complex as a biomimetic sensor material. Analysis of hydrogen peroxide in the excised plant material using novel AuND-based electrochemical cells and modified glassy carbon (GC) electrodes is compared with the analysis using a spectrophotometric method and reveals a good correlation of the results. The increased level of hydrogen peroxide due to abiotic stress (intense sunlight) with the proposed electrochemical system is also demonstrated by *in vivo* real-time detection.

## Materials and Methods

### Chemicals

Dimethylformamide (DMF), trichloracetic acid (TCA), horseradish peroxidase (HRP) (type VI, ≥250 units/mg), catalase (from bovine liver, 2,000–5,000 units/mg), 4-aminoantipyrine (4-AP), phenol, graphene oxide (GO), activated charcoal, agar powder, potassium chloride, silver nitrate, potassium hexachloroplatinate (IV), gold(III) chloride trihydrate, aceton, 2-isopropanol, ethanol, and other chemicals were purchased from Sigma-Aldrich (St. Louis, United States). All reagents were of analytical grade. Mn(III) *meso*-tetra(N-methyl-4-pyridyl) porphine pentachloride (MnTMPyP) and Mn(III) *meso*-tetra(4-pyridyl) porphine chloride (MnTPyP) (>95%) were obtained from Frontiers Scientific Inc. Phosphate buffer (PB) solutions (0.1 M, pH 7.4) were prepared from disodium hydrogen phosphate and sodium dihydrogen phosphate. The pH of the solutions was controlled by the pH-meter 765 (Knick GmbH). All solutions were prepared using a Milli-Q water purification system (Merk KGaA, Germany).

### Methods and Apparatus

Two types of electrodes were used: thin-film gold electrodes and AuND electrodes prepared by DENA.

The thin-film gold electrodes (Si/SiO_2_ 1 µm/Ti 10 nm/Au 400 nm, 11 mm × 11 mm) were prepared using vapor phase deposition on a Balzers (Pfeiffer) PLS 500 system for thin film deposition in an ISO 5 cleanroom and cleaned as described in (Liu et al., 2015). The surface of the thin-film gold electrodes that was exposed in the electrochemical cell for contact with the electrolyte solution amounted to 0.196 cm^2^.

**FIGURE 1:**
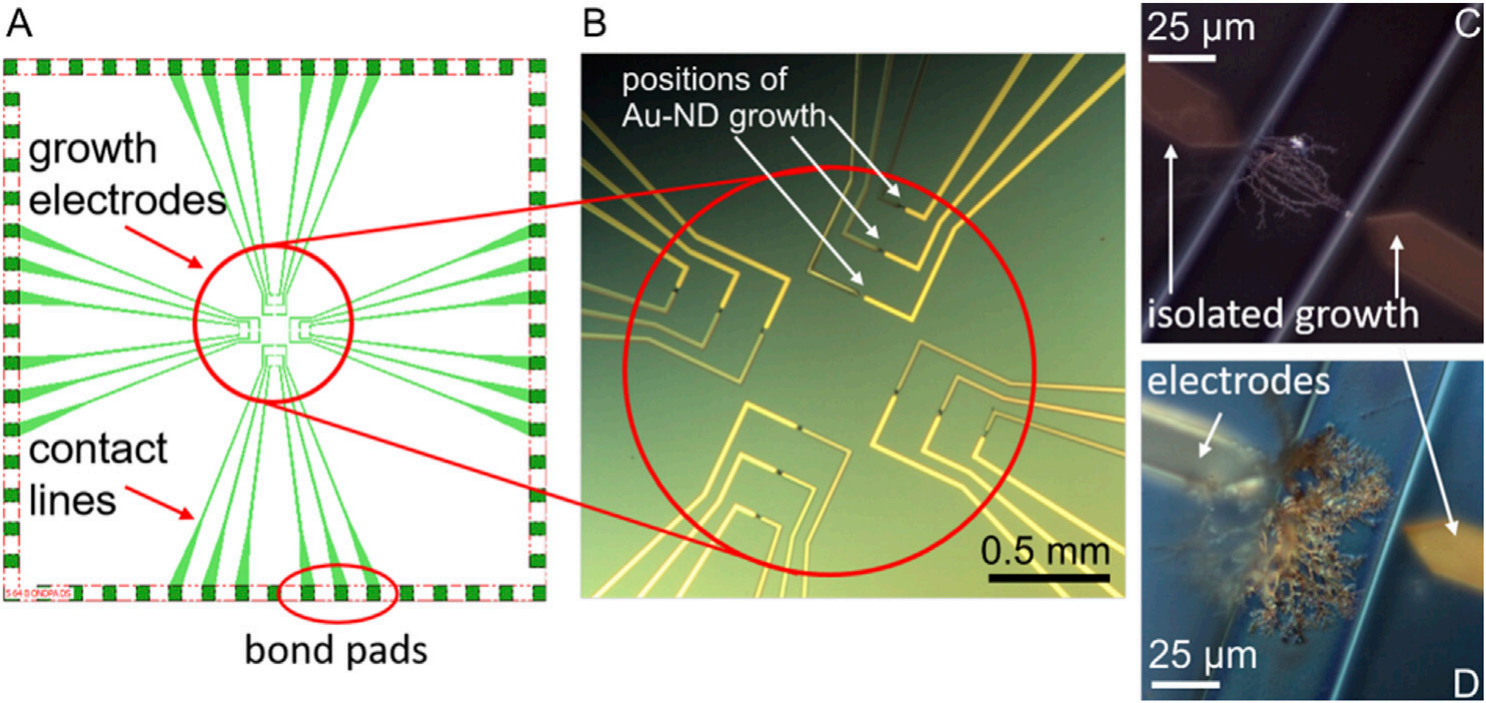
The chip design (not scaled) **(A)** and optical microscopy image **(B)** of the electrochemical system with the AuND electrode growth area (circled in red), white arrows show examples of the growth positions of the AuND. Examples of the AuND electrodes after isolation **(C,D)**. The electrode gap between the growth electrodes is 50 µm.

For DENA, the Si/SiO_2_ 1 µm chips (13 mm × 13 mm) with the thin-film growth electrodes, contact lines, and bond pads of Ti 10 nm/Au 100 nm were prepared in an ISO 5 cleanroom as described in previous works (Nikolaev et al., 2018a; Nikolaev et al., 2018b). The chip design is shown in Figures 1A,B. Briefly, a boron-doped n-Si (111) wafer was oxidized to produce a silicon dioxide layer of 1 µm thickness using a Tempress oxidation furnace. This Si/SiO_2_ wafer was further used as a carrier for the deposition of the growth microelectrode circuitry by photolithography and lift-off processes. After dehydration at 180°C for 20 min, the Si/SiO_2_ wafer was coated with a LOR 3B photoresist to produce a layer of 5 μm, baked for 5 min, and subsequently coated with an AZ nLOF 2020 photoresist to produce a layer of 2 µm. A photoresist stack was used to achieve a better control over the geometry of the growth microelectrodes and contact lines. The coated wafer was pre-baked on a hot plate at 115˚C for 90 s. The Si/SiO_2_ wafer with a photoresist was exposed at 325 W using a photolithography mask at Mask Süss MA-6 (Hg-vapour lamp 350 W). Exposure time was optimized as 1.4 s. After exposure, the wafer was post-exposure baked at 115˚C for 90 s and developed by AZ® 326 (MIF, 2.38% TMAH in H_2_O) for 1 min to produce structured photoresist layers for subsequent metallization and lift-off processes. Ti (10 nm) and Au (100 nm) were deposited on the Si/SiO_2_ wafer with pre-structured photoresist using an electron beam evaporation. After metallization, the wafer was lifted off with acetone and cleaned in isopropanol and distilled water.

The *in situ* electrochemical synthesis of the AuND electrodes on these chips by DENA and their subsequent isolation, Figures 1C,D are described in detail (Nikolaev et al., 2017; Nikolaev et al., 2018b). Briefly, the growth electrodes obtained as described above were connected in pairs to a high frequency generator (Agilent TrueformSeries Waveform Generator, 33,600 Series, High Frequency Model) of direct and alternating voltages. One of the electrodes served as a voltage source and the other as a drain electrode, Figure 1B. High frequency alternating voltage was generated as a square-wave frequency step with a frequency of 45 MHz. The applied alternating voltage was typically 18.5 V and direct voltage +1 V. Nanowires were synthesized using a 5 µl volume of an aqueous solution of HAuCl_4_⋅3H_2_O. The growth electrodes and contact lines were isolated by polyimide polymer via a photolithography process, Figure 1C,D. After photolithography, only channels with the DENA-grown nanodendrite electrodes were open for contact with the electrolyte solutions and bond pads were open for contact to the external circuitry. The contact pads of the electrodes were connected with wires using conductive silver epoxy (EPO-TEK H20E, Epoxy Technology, Inc.). Finally, the chips were isolated with an epoxy glue (UHU GmbH, Germany) or EPO-TEK 302-3M (Epoxy Technology, Inc.). For the measurements and electrode modifications in the electrolyte solutions, a glass ring (radius 8 mm) was adapted to the chips, leaving the bond pads outside the ring.

The electrochemical reduction and deposition of graphene oxide (1 mg ml^−1^) on gold electrodes was performed in 0.1 M LiClO_4_ solutions with pH 3.6 adjusted by HClO_4_. The deposition was carried out for 6 min in the deaerated solutions. Mn(III) porphyrin complexes were adsobed from the 8 × 10^–5^ M porphyrins in DMF. Modifications of the gold nanodendrite (AuND) electrodes to prepare a counter Pt-nanodendrite electrode and an Ag/AgCl reference electrode on a chip were carried out by applying the corresponding reduction or oxidation potentials to the AuND electrodes in solutions of 0.01 M K_2_[PtCl_6_] in 0.01 M HClO_4_ (−2 V against Pt wire), 0.02 M AgNO_3_ in 0.01 M HNO_3_ (−1 V against Ag wire), and 0.01 M KCl (1 V against Pt wire) using a TDK-Lambda power supply (TDK-Lambda Germany GmbH).

Electrochemical measurements and GO deposition were performed using a three-electrode cell controlled by an Autolab PGSTAT (Metrohm, The Netherlands) and Nova 2.1 software. A coiled platinum wire electrode and an Ag/AgCl double-junction reference electrode (Metrohm, Switzerland) were used as counter and reference electrodes, respectively. If required, the solutions were deaerated with argon.

The electroactive surface coverage of the MnP complex on the electrodes was found using the formula (Koposova et al., 2013): Γ = *Α*/nFAυ, where Γ is the surface coverage of a porphyrin complex on the electrode surface, *A* is the redox current peak area, A is the surface area of an electrode, F is the Faraday constant, n is the number of electrons transferred (*n* = 1), and υ is the scan rate. The surface area of the AuND electrodes was found as described in (Nikolaev et al., 2015) using the cyclic voltammograms in a potential range of 0–1.5 V and the formula Γ = A/(ν⋅482 μC cm^−2^), where Γ is the electroactive area, ν is a scan rate, and 482 μC cm^−2^ is the charge density per unit area for the electrochemical reduction reaction of a monolayer of chemisorbed oxygen on polycrystalline gold, and Α is a cathodic peak area in the cyclic voltammograms.

A solar light simulator device with a 150 W xenon arc lamp, an air mass filter AM 1.5, and a 390–650 nm visible light filter was used for the experiments (Oriel Instruments). The device can be operated at different light intensities given by a lamp operation power of 150 and 95 W. The light intensity at 95 W corresponded to 1000 W m^−2^, as measured by a photodetector (CAS140CT-154 compact-array-spectrometer model UV−vis−NIR, Instrument Systems).

Spectrophotometric measurements were performed using a PerkinElmer Lambda 900 spectrometer and quartz cuvettes with a path length of 0.5 cm. Scanning electron microscopy (SEM) characterization and energy-dispersive X-ray spectroscopy (EDX) analysis of the electrodes were performed using a Magellan XHR SEM system equipped with an EDX detector.

### Determination of Hydrogen Peroxide in Plant Tissue and *In Vivo* Monitoring of Hydrogen Peroxide in Plant Leaf

Analysis of hydrogen peroxide in plant materials was performed using excised leaf disk after sample preparation as well as *in vivo* monitoring of hydrogen peroxide in plant leaf using the developed sensor system was tested. In the first case, leaf disks (2.5 g) were excised and immediately frozen in liquid nitrogen for 3–4 min. The frozen plant material was ground in a mortar together with a cold 5% TCA (8 ml, 4°C) and activated charcoal (0.5 g) on ice. The mixture was centrifuged (10,000 g for 20 min at 4°C) and the supernatant was immediately filtered (0.45 µm, Millipore) using a cold syringe and collecting flask. The filtrate was adjusted to pH 7.4 with 2 M KOH and the extent of dilution was noted. The solution was filtered again to remove some white precipitate which was formed after neutralization (probably calcium oxalate) (Patterson et al., 1984). Blanks were prepared by removing hydrogen peroxide from the neutralized samples with catalase (1 μg ml^−1^, 10 min) followed by the addition of a colorimetric agent after 10 min. The exact same sample preparation time was used for all plant samples.

Colorimetric detection based on the Trinder method was used, which utilizes 4-AP and phenol as a donor substrate to reduce hydrogen peroxide in the presence of peroxidase according to the following reaction scheme (Frew et al., 1983; Varadaraju et al., 2018):

$$2H_{2}O_{2}+4-AP+phenol\to^{HRP}colored\;product+4\;H_{2}O$$ (1)

The resulting quinoneimine colored product has an absorption maximum at 505 nm.

The colorimetic reagent solution (10 ml) contained 10 mg 4-AP, 15 mg phenol, 1.25 mg HRP, and 1 ml of 0.1 M PB. A 1 ml aliquot of the sample solution was mixed with 0.9 ml of the colorimetric reagent and the absorbance was scanned over the wavelength range 520–490 nm after 12 min at room temperature. The method of standard additions was also used to quantify the concentration of hydrogen peroxide in the plant samples. When using the method of standard additions, the plant samples were spiked with the known amount of hydrogen peroxide. The data were analyzed statistically using the Student’s t-test.

For the *in vivo* experiments with a plant leaf cut surface, a thin layer of the agarose gel was applied to the edges of the isolated chips without a glass ring to facilitate contact between the chip and the plant leaf. The gel was prepared by mixing 0.1% w/v agar powder with 0.1 M PBS (pH 7.4) at 90°C. A thin upper layer of the leaf was cut with a scalpel. A 30 µl drop of the electrolyte solution (0.1 M PB with 0.05 M KCl) was applied to the chip electrode area to improve contact with a leaf cut surface. The amperometric measurements were performed directly after the contact of the chip with the leaf cut surface, after illumination with a solar light illumination device at 95 W for 30 min, and after addition of catalase, 5 µl of approx. 1 μg ml^−1^.

## Results and Discussion

### Modification of Gold Electrodes With ErGO and MnP Complex

**FIGURE 2:**
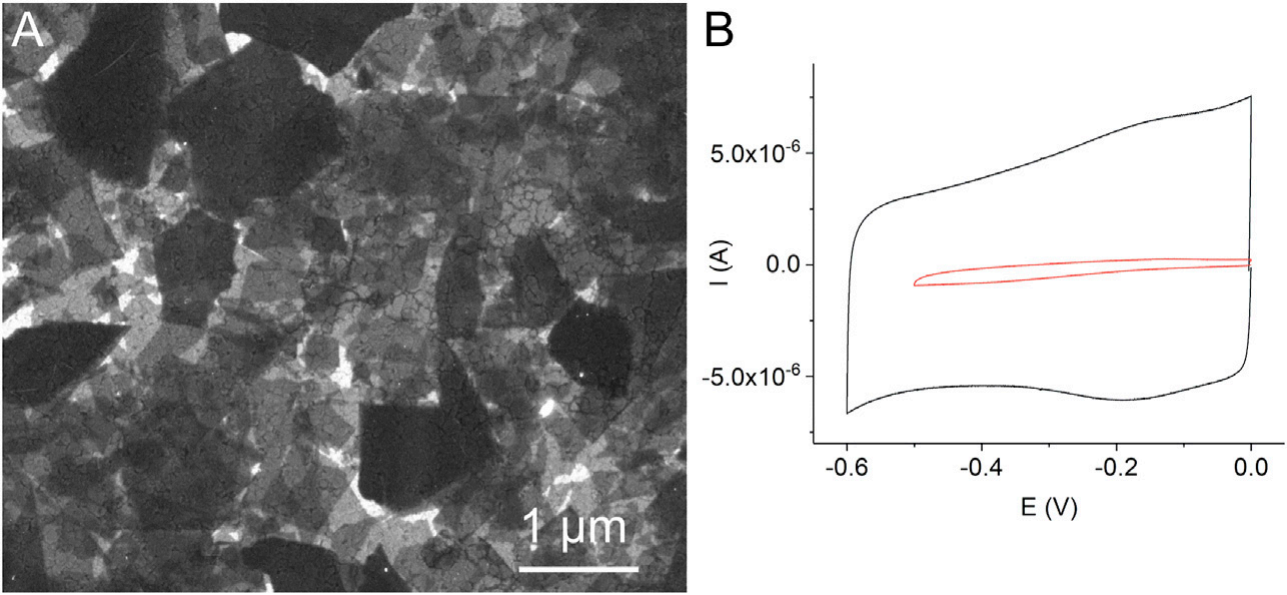
A SEM image of the thin-film gold electrode with the electrochemically reduced graphene oxide **(A)**. Cyclic voltammograms of a bare gold electrode (red line) and a gold electrode modified with the electrochemically reduced graphene oxide (black line) in 0.1 M PB, scan rate 0.05 Vs^−1^, deaerated solutions **(B)**.

**FIGURE 3:**
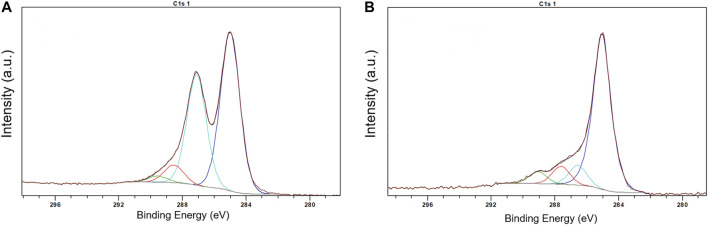
XPS spectra of GO **(A)** and ERGO **(B)**.

Preliminary experiments showed that the method of the direct adsorption of MnTMPyP and MnTPyP on the thin-film gold electrodes was not effective for the preparation of the hydrogen peroxide sensors since these porphyrins were insufficiently adsorbed on the bare gold electrode surface, Supplementary Figure S1. Cyclic voltammogram of a thin-film gold electrode after adsorption of the MnTMPyP complex revealed no redox processes of the porphyrin complex, as it is exemplarily shown on Supplementary Figure S1. Recently, the electrochemical deposition of the electrochemically reduced graphene oxide (ErGO) on the carbon fiber (Bai et al., 2016) and stainless steel (Farajikhah et al., 2019) electrodes has been used to create an interface for the electrochemical sensors. Metalloporphyrin complexes interact and adsorb well on carbonaceous materials and GCEs. Figure 2A shows a SEM image of the thin-film gold electrode surface, which was modified with the ErGO. In addition, cyclic voltammetry reveals a higher capacity of the thin-film gold electrode modified with ErGO in comparison with a bare gold electrode, Figure 2B. Additionally, Figures 3A,B show the C1s XPS spectra of the GO and electrochemically reduced ErGO on the thin film gold electrodes, respectively. One can see that after electrochemical reduction, Figure 3B, the peaks associated with C-C (285.0 eV) became predominant, while the peaks associated with the oxidized carbon species, C-O (286.6 eV), C=O (287.6 eV), and O-C=O (289.1 eV), were greatly weakened. This result reveals that the oxygenated functional groups were efficiently reduced during the electrochemical reduction and is in agreement with previous study (Bai et al., 2015).

**FIGURE 4:**
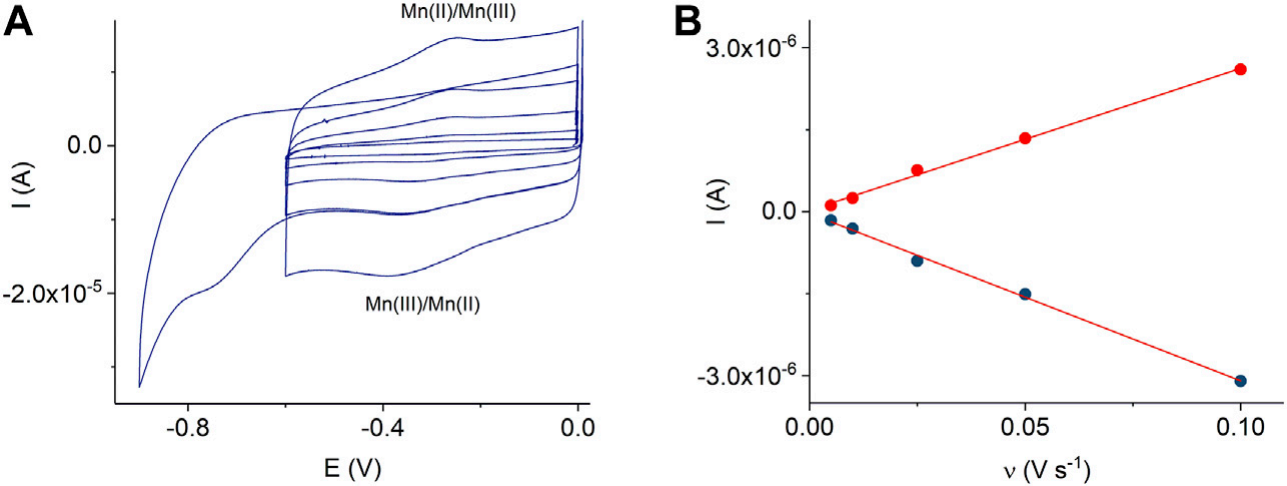
CVs of the adsorbed MnTMPyP complex on the thin-film Au/ErGO electrode recorded in a deaerated 0.1 M PB, pH 7.4, scan rate from 0.005 Vs^−1^ to 0.1 Vs^−1^ **(A)**. Dependencies of the Mn(III/II) peak currents on the scan rate, R^2^_oxidation_ = 0.9964 and R^2^_reduction_ = 0.9965 **(B)**.

A comparison of Figure 4 and Supplementary Figure S1 shows that the MnTMPyP complex is adsorbed much better on the electrode surface modified with the ErGO than on a bare gold surface**.** In the former case, clearly distinguishable redox currents of the immobilized porphyrin complex are observed, Figure 4A. In the deoxygenated solutions, cyclic voltammogramms reveal the electrochemical reduction, E_p_^Mn(III)/(II)^ = −0.329 V, and oxidation, E_p_^Mn(II)/Mn(III)^ = −0.258 V, ΔE = 0.71 V (at a scan rate of 0.005 V s^−1^), of the central metal ion in the coordination center of the metalloporphyrin, which is essential for the electrocatalytic conversion of H_2_O_2_ in subsequent experiments (Peng et al., 2020). At about E_p_^L, red.^ = −0.760 V, an irreversible electrochemical reduction of the porphyrin ligand occurs, Figure 4A. The Mn(III)/(II) redox potential and peak separation values are slightly different from the redox potential values of the same porphyrin complex in solutions or adsorbed on a GCE, while E_p_^L, red.^ is close to the corresponding value in the solution (Mourzina and Offenhäusser 2020). This can be explain by the change in the orientation and environment of the central metal ion due to adsorption, while functional groups of the electrode surface can assume the role of the axial ligands. Dependencies of the reduction and oxidation peak currents on the scan rate confirm the adsorption behavior, Figure 4B.

However, the immobilization method used in this study was unsuccessful in immobilizing another complex, MnTPyP, on the ErGO interface, Supplementary Figure S2, with virtually no sensitivity to hydrogen peroxide in the concentration range investigated. It is possible that the positively charged N-methyl-4-pyridyl groups of the MnTMPyP complex interact with some negatively charged oxygen-containing ErGO groups, which promotes a better adsorption of the MnTMPyP complex compared to MnTPyP. Therefore, MnTPyP was not used any further for the development of the electrochemical system in this study.

The electroactive surface coverage of the MnTMPyP complex on the Au/ErGO electrodes was found as described in the experimental section and amounted to (2.4 ± 0.6) 10^–10^ mol cm^−2^. This value is about 10 times higher than that on a GCE and other surfaces (Peng et al., 2020), which can be explained by the increase in the effective electrode surface area due to the ErGO nanomaterial. The Mn(III)/(II) heterogeneous electron transfer constant determined by the Laviron method (Laviron 1979) amounted to (0.3 ± 0.08)s^−1^. Furthermore, unlike adsorption on the GCE (Peng et al., 2020), MnTMPyP is more stable on the ErGO. Thus, electrochemical deposition of the GO on the gold electrodes can be used to create the ErGO interface for the subsequent deposition of high amounts of the electroactive MnTMPyP complex on the modified surfaces. This method was used further for the modification of the AuND electrodes to prepare working electrodes for the electrochemical system.

## On-Chip Electrochemically Assembled Sensor System

**FIGURE 5:**
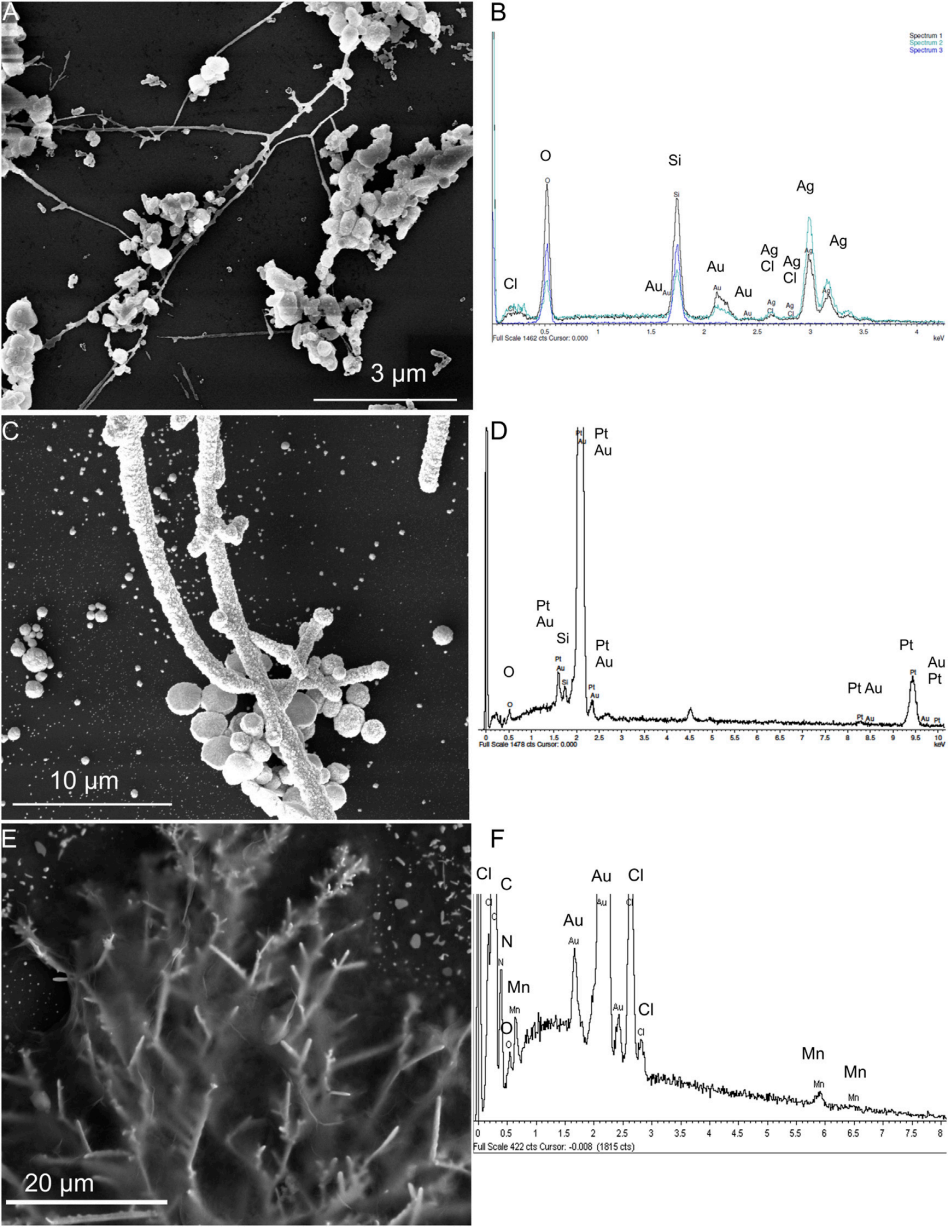
SEM images and EDX spectra of the Ag/AgCl reference electrode **(A,B)**, Pt counter electrode **(C,D)** electrochemically deposited on the Au-ND electrodes, and ErGO/MnTMPyP working electrode **(E,F)** of the on-chip electrochemical system based on the Au-ND electrodes.

In the on-chip electrochemical sensor system, the three-electrode system consisted of an Ag/AgCl reference electrode, a Pt counter electrode, and an ErGO/MnTMPyP working electrode, which were prepared on the DENA-assembled AuND electrode platform by electrochemical depositions, as described in the experimental section. Figures 5A–F shows SEM images and EDX spectra of three electrodes of the on-chip electrochemical sensor system. EDX analysis confirmed the composition of the modified nanodendrite electrodes.

**FIGURE 6:**
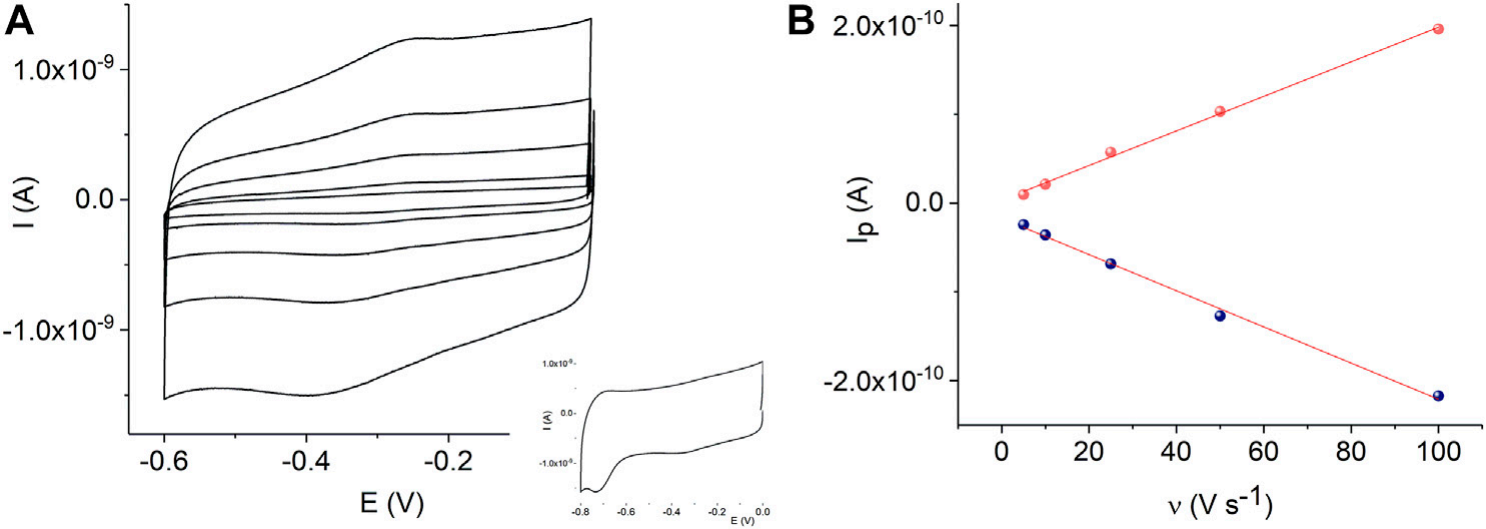
CVs of the adsorbed MnTMPyP complex on the AuND/ErGO electrode recorded in a deaerated 0.1 M PB, pH 7.4, scan rate from 0.005 Vs^−1^ to 0.1 Vs^−1^, insert shows reduction of the porphyrin ligand **(A)**. Dependencies of the Mn(III/II) redox peak currents on the scan rate, R^2^_oxidation_ = 0.9969 and R^2^_reduction_ = 0.9952 **(B)**.

In addition, Figure 6A shows cyclic voltammograms of the MnTMPyP complex adsorbed on the AuND/ErGO electrodes in the deoxygenated solutions. Surface confinement of the MnP is confirmed by the linear dependence of the redox peak currents on the scan rate, Figure 6B. Cyclic voltammogramms reveal the electrochemical reduction at E_p_^Mn(III)/(II)^ = −0.332 V, and oxidation, E_p_^Mn(II)/Mn(III)^ = −0.255 V, ΔE = 0.77 V (at a scan rate of 0.005 V s^−1^), of the central metal ion in the coordination center of the metalloporphyrin. These values are similar to the redox potentials of the MnTMPyP on the thin-film Au/ErGO electrodes, while the Mn(III)/(II) heterogeneous electron transfer constant determined using the Laviron method has a lower value (0.09 ± 0.02) s^−1^. The surface area of the AuND electrode and electroactive surface coverage of the MnTMPyP complex on the AuND/ErGO electrodes were found as described in the experimental section and electroactive surface coverage of the MnTMPyP amounted to (1.7 ± 0.5) 10^–10^ mol cm^−2^, which is similar to the electroactive surface coverage found for the thin-film electrodes above.

**FIGURE 7:**
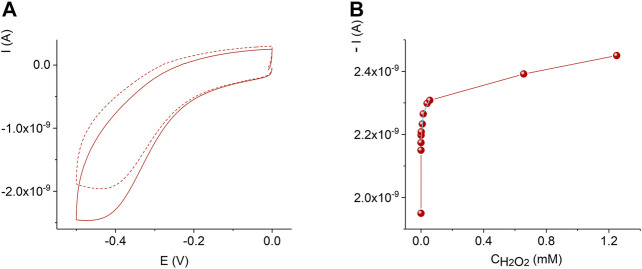
Cyclic voltammograms of the AuND/ErGO/MnTMPyP electrochemical system with 0 M (dashed line) and 1.2 mM (solid line) H_2_O_2_ **(A)**. Dependence of the current on the concentration of hydrogen peroxide at -0.45 V for the AuND/ErGO/MnTMPyP **(B)**. Other conditions: 0.1 M PBS, pH 7.4, scan rate 0.05 Vs^−1^, measurements were performed at ambient conditions.

Figure 7 displays the sensor response and calibration curve of the AuND/ErGO/MnTMPyP sensor system in solutions with different concentrations of hydrogen peroxide. For comparison, Supplementary Figure S3 shows the response of the thin-film Au/ErGO macroelectrode without the MnP complex in similar conditions. The thin-film Au/ErGO macroelectrode without the MnP complex, Supplementary Figure S3, reveals practically no sensitivity to hydrogen peroxide, which emphasizes the importance of the MnTMPyP complex for the hydrogen peroxide sensing properties of the system.

Comparing Figures 6A,7A as well as Supplementary Figure S4, one can see that in the presence of oxygen and hydrogen peroxide electrochemical reduction and oxidation processes of the central metal ion, Mn^II/III^P, were not observed, while the cathodic current increased. Electrocatalytic reduction of oxygen and hydrogen peroxide starts at potential close to the potential of the Mn(II/III) redox couple. Accordingly, the electrochemically generated metal-reduced forms, Mn(II)TMPyP, are involved in the reaction with oxygen and hydrogen peroxide oxidants and regenerated due to electrocatalyst redox cycling according to a number of proposed mechanisms (Baglia et al., 2017; Mourzina and Offenhäusser 2020). Moreover, previously, we found that for the MnTMPyP electrocatalyst in solution the electrocatalytic reduction currents were higher in the presence of oxygen than in the deaerated conditions for the same concentration range of hydrogen peroxide (Mourzina and Offenhäusser 2020), Supplementary Figure S4. This behavior can be explained by a number of parallel processes with participation of superoxide radical anion produced from the reduction of oxygen (Mourzina and Offenhäusser 2020) such as, e.g., promoting a cleavage of the OO bond of hydrogen peroxide axially ligated to a reduced Mn(II) porphyrin complex, so that due to the synergy of electrocatalytic effect of MnTMPyP complex and oxygen higher reduction currents and higher sensitivity to hydrogen peroxide are observed at ambient conditions. Similarly, we found that in the case of the immobilized MnTMPyP, the electrocatalytic reduction currents and sensitivity to hydrogen peroxide were higher at ambient conditions (in the presence of oxygen) (Peng et al., 2020), which is similar to the experiments with the MnTMPyP complex in the solutions. Thus, the response of the sensor based on the MnTMPyP redox cycling can be described by Scheme 2, although other processes that are mentioned above may contribute to the sensor response, which require further studies. It is assumed that the reaction proceeds via metal-oxo intermediates similar to the iron porphyrins (Baglia et al., 2017).

**SCHEME 1:**
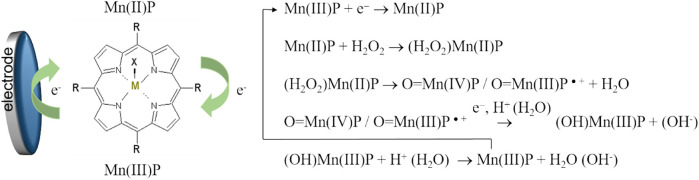
Scheme of the electrocatalytic reduction of hydrogen peroxide on the electrode modified by the MnTMPyP complex.

The AuND/ErGO/MnTMPyP sensor system response at the detection potential of −0.45 V, Figure 7B, can be approximated with two linear intervals: 3.4⋅10^–7^ M—1.5⋅10^–5^ M with a sensitivity of 0.384 A M^−1^ cm^−2^ found from the linear regression equation with *R* ^2^ = 0.79996, and 1.5⋅10^–5^ M—8⋅10^–5^ M with a sensitivity of 0.07 A M^−1^ cm^−2^ found from the linear regression equation with *R* ^2^ = 0.9157. The detection limit was found as 3.3⋅S/b, where b is the slope of the calibration curve in a low concentration range and S is the standard deviation of the blank sample measurements (ICH Harmonized tripartite guideline, 2015), and was as low as (3.2 ± 0.3)⋅10^–7^ M H_2_O_2_. It is important that the detection of low concentrations of hydrogen peroxide at the negative potential can be performed in the presence of oxygen (Mourzina and Offenhäusser 2020), while some reported electrochemical sensors can only be used in the deoxygenated solutions.

### Determination of Hydrogen Peroxide in Plant Tissue by Spectrophotometric and Electrochemical Methods

**FIGURE 8:**
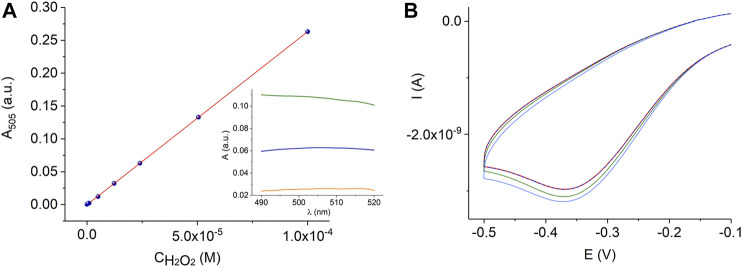
Calibration graph for the peroxidase-coupled reaction according to reaction scheme (1) in 0.06 M PB, pH 7.4, *R* ^2^ = 0.9998, where *R* ^2^ is the coefficient of determination of the linear regression. The insert shows the absorbance spectra of a quinoneimine product of the colorimetric reaction of 2.4⋅10^–5^ M H_2_O_2_ in PB (blue line) and plant leaf extracts (sunflower, *Helianthus annuus* L., - orange line and *Kalanchoe blossfeldiana* - green line) **(A)**. Electrochemical measurements with the AuND/ErGO/MnTMPyP electrochemical system in the wheat plant extracts (dashed red line) with additions of hydrogen peroxide (green and blue lines) **(B)**.

Sample preparation for the analysis of hydrogen peroxide in plant tissue is a major challenge due to the decomposition of hydrogen peroxide and the reduction of hydrogen peroxide by the sample components. Moreover, the hydrogen peroxide content in plant material fluctuates at different times of the day (Cheeseman 2006) depending on the excised part of the leaf and many other factors. The extraction protocols and assay methods of hydrogen peroxide in plant material have not yet been fully established (Alexieva et al., 2001; Cheeseman 2006; Veljovic-Jovanovic et al., 2002). Moreover, living plant tissues contain high activities of catalases and peroxidases, which must be inactivated before hydrogen peroxide can be extracted. It was shown that plant cells can completely metabolize as high as 10 mM H_2_O_2_ in less than 10 min (Cheeseman 2006). Therefore, the liquid nitrogen freezing of samples followed by TCA treatment is often employed in many sample preparation methods because TCA is an excellent protein precipitant and deactivates enzymes that otherwise destroy hydrogen peroxide during sample preparation. Activated charcoal is also used to remove colored carotinoides and other plant pigments, phenolic compounds, pheophytin*,* and ascorbic acid, which is a hydrogen peroxide reducing agent. In this study we used protocols for the determination of hydrogen peroxide in plant material described in (Kreslavskii et al., 2011; Patterson et al., 1984; Zhou et al., 2006) and spectrophotometric quantification based on the Trinder method, Figure 8A, or electrochemical sensor measurements, Figure 8B, as described in the experimental section, Table 1. To prove the results obtained with the AuND/ErGO/MnTMPyP system, we additionally used electrochemical quantification using GCE/MnTMPyP macrosensors described in (Peng et al., 2020), Supplementary Figure S5 and Table 1. Table 1 compares the results of the analysis of the plant material using spectrophotometric and electrochemical quantification. The results of both methods are comparable with the hydrogen peroxide content reported by other authors (Ranieri et al., 2003; Agüera et al., 2010; Si et al., 2018).

**TABLE 1:** Analysis of the content of hydrogen peroxide in leaves of various plant species using spectrophotometric and electrochemical quantification (*p* = 0.95, *n* = 5).

	Detection method		
Plant species	Spectrophotometric, mol (g FW)^−1^	Electrochemical with GCE/MnTMPyP^a^, mol (g FW)^−1^	Electrochemical system with AuND/ErGO/MnTMPyP, mol (g FW)^−1^
Sunflower, *Helianthus annuus* L	(8.8 ± 0.5)⋅10^–8^	(9.7 ± 0.9)⋅10^–8^	(9.4 ± 1.1)⋅10^–8^
Wheat, *Triticum aestivum* L	(1.1 ± 0.1)⋅10^–7^	(1.6 ± 0.2)⋅10^–7^	(1.8 ± 0.2)⋅10^–7^
*Kalanchoe blossfeldiana*	(3.5 ± 0.2)⋅10^–7^	(4.0 ± 0.3)⋅10^–7^	(3.1 ± 0.4)⋅10^–7^

^a^ The GCE/MnTMPyP electrodes were from Peng et al. (2020).

### *In Vivo* Monitoring of Hydrogen Peroxide in Plant Tissue With AuND/ErGO/MnTMPyP Electrochemical System

**FIGURE 9:**
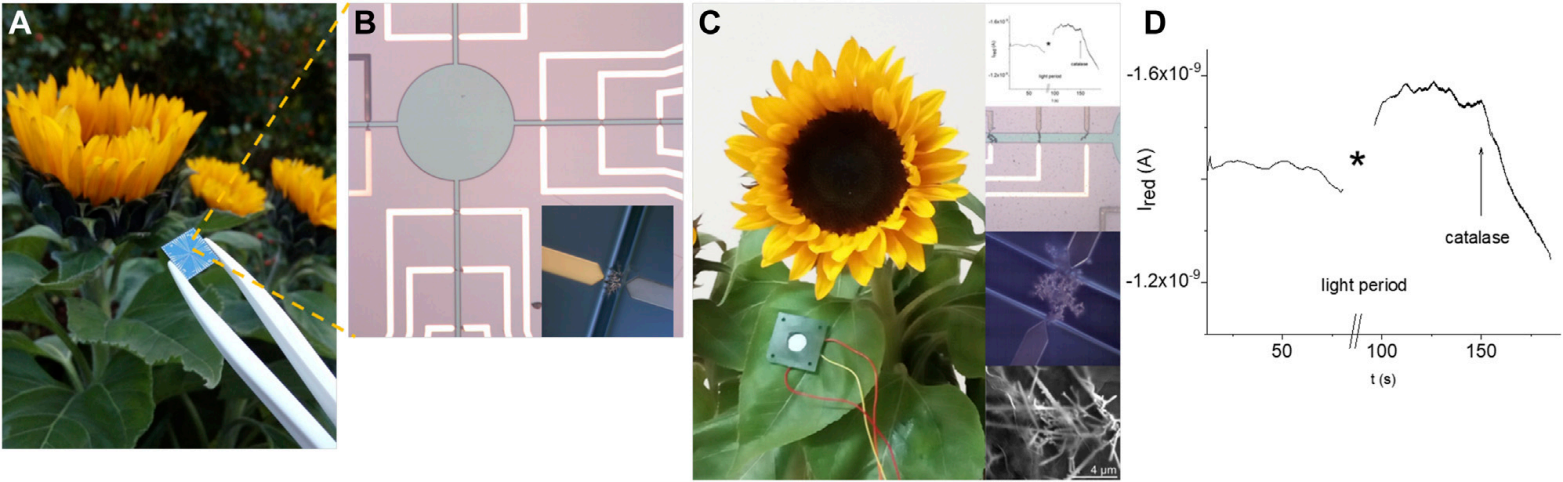
Feasibility of the real time *in vivo* monitoring of the hydrogen peroxide reduction current in a cut leaf surface of the sunflower using an electrochemical sensor system: actual image of a chip in comparison with a sunflower **(A)**, area of the sensor system with electrodes and opening (circle and channels) for the contact with liquid media (insert displays an electrode in the open channel) **(B)**, example of the positioning of the electrochemical system with the amperometric response before irradiation with light, after irradiation with light, and the addition of catalase (inserts show fragments of the electrochemical system and amperometric measurements) **(C,D)**.

Figure 9 displays the real-time *in vivo* amperometric monitoring of the reduction currents of hydrogen peroxide in the cut leaf surface of the sunflower using the electrochemical system before and after sun light illumination for 30 min. The increased reduction current indicates the generation of hydrogen peroxide, while the addition of catalase results in the decrease of the reduction current due to the induced decomposition of H_2_O_2_.

## Conclusion

In this study, we demonstrated a method for the directed electrochemical nanowire assembly of a sensor system for the determination of hydrogen peroxide. It was shown that the electrochemical reduction and deposition of graphene oxide on the gold electrode surfaces enable an efficient sensor interface for the adsorption of a biomimetic electrocatalytic sensor material, Mn(III) *meso*-tetra(N-methyl-4-pyridyl) porphyrin complex, with as high as 10^–10^ mol cm^−2^ surface coverage. The electrochemical sensor system is characterized by a low limit of detection (0.3 µm H_2_O_2_) in the presence of oxygen. The novel sensor system was successfully applied for the electrochemical determination of hydrogen peroxide in plant leaves and *in vivo* real-time monitoring of the hydrogen peroxide dynamics as a sign of abiotic stress (intense sunlight). The results are in good agreement with the results of the biochemical analysis in which spectrophotometric detection is based on the Trinder method. The novel electrochemical sensor platform might help with studies on the mechanisms of plant response to stress factors as well as with understanding the complex ROS plant signaling network. We anticipate that this method can be extended for synthesis and integration of multisensor arrays in analytical microsystems and devices to determine other analytes and biomarkers.

## Data Availability

The original contributions presented in the study are included in the article/Supplementary Material, further inquiries can be directed to the corresponding author.
