# Supplementary material for: Synthesizing Electrodes Into Electrochemical Sensor Systems
Source: Front Chem. 2021 Mar 31;9:641674. doi: 10.3389/fchem.2021.641674 (PMC8044375; doi:10.3389/fchem.2021.641674)
Supplement: Supplementary file 1 [file datasheet1.docx]

***SUPPORTING INFORMATION***

**Synthesizing electrodes into electrochemical sensor systems.**

Yulia G. Mourzina^1*^, Yuri E. Ermolenko^2^, Andreas Offenhäusser^1^,

^1^Institute of Biological Information Processing - Bioelectronics (IBI-3), Forschungszentrum Jülich, 52425, Jülich, Germany

^2^Institute of Chemistry, St. Petersburg State University, Universitetskaya nab. 7-9, 198504, St. Petersburg, Russia

*Corresponding authors: [y.mourzina@fz-juelich.de](mailto:y.mourzina@fz-juelich.de) (Dr. Yu. Mourzina)





**SUPPORTING FIGURE S1.** Cyclic voltammogram of a thin-film gold electrode after adsorption of the MnTMPyP complex from the DMF solution, 0.1 PB pH 7.4, scan rate from 0.005 to 0.2 V s^-1^, deaerated solutions.





**SUPPORTING FIGURE S2.**  CVs of the adsorbed Mn(III) *meso*-tetra(4-pyridyl) porphine, MnTPyP complex, on an Au/ErGO thin-film electrode recorded in a deaerated 0.1 M PB, pH 7.4, scan rate from 0.005 V s^-1^ to 0.1 V s^-1^.


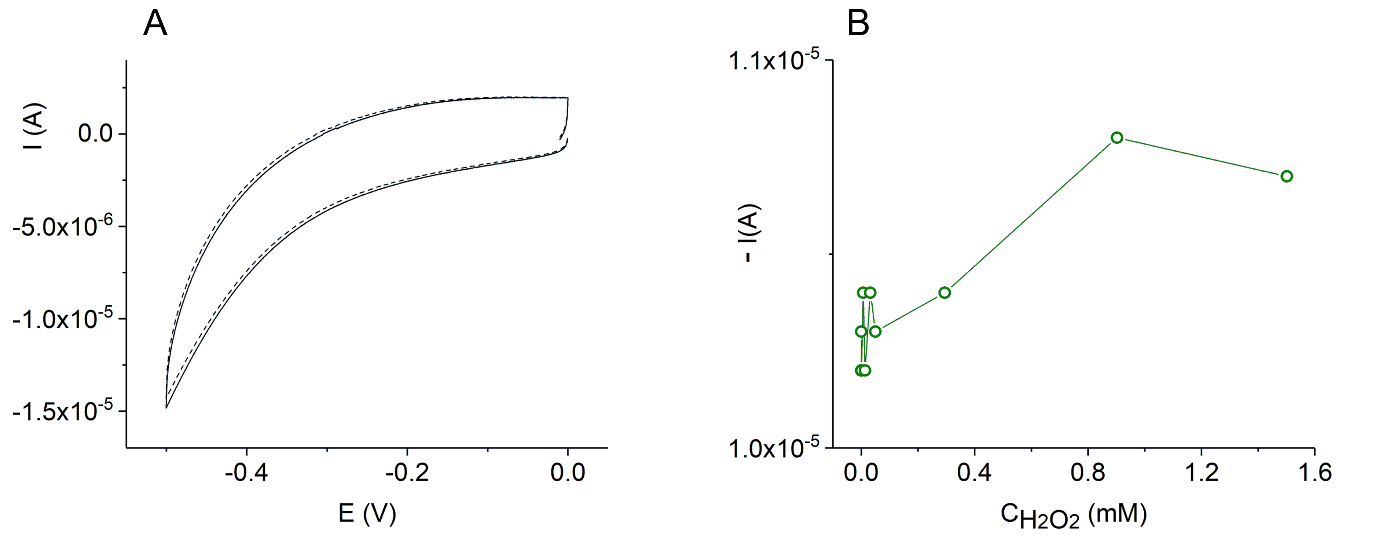


**SUPPORTING FIGURE S3.** Cyclic voltammograms of the thin-film Au/ErGO macroelectrode with 0 M (dashed line) and 1.5 mM (solid line) H_2_O_2_ **(A)**. Dependence of the current on the concentration of hydrogen peroxide at -0.45 V for the thin-film Au/ErGO macroelectrode **(B)**. Other conditions: 0.1 M PBS, pH 7.4, scan rate 0.05 V s^-1^, measurements were performed at ambient conditions.

**
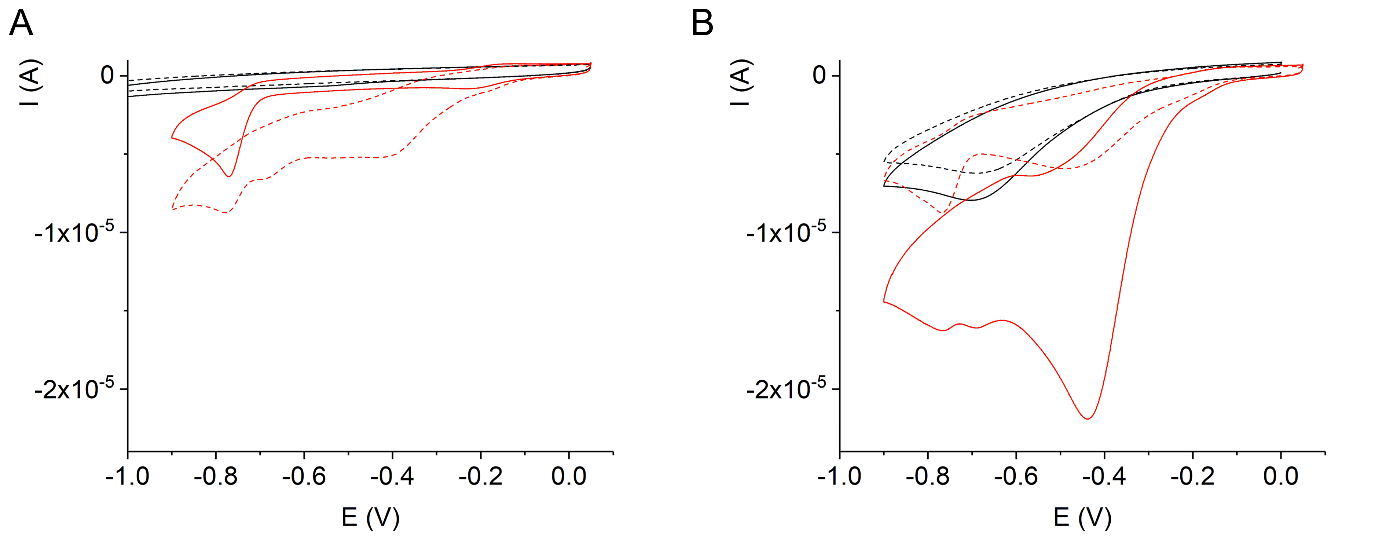
**

**SUPPORTING FIGURE S4.** Cyclic voltammograms demonstrating the electrocatalytic effect of the MnTMPyP complex in the hydrogen peroxide reduction in the deoxygenated solutions **(A)** and at ambient conditions (in the presence of oxygen) **(B)**. The scale in **(A)** and **(B)** is made the same to facilitate the comparison of the results. Black lines correspond to the GCE without a manganese porphyrin complex in the solution, red lines - with MnTMPyP complex in solution at a concentration of 9⋅10^-5^ M, dashed lines - 0 M H_2_O_2_, and solid lines - 3.4⋅10^-3^ M H_2_O_2_. Hydrogen peroxide electroreduction starts at more positive potentials (at the lower energy on the energy scale) in the presence of the MnTMPyP complex in the solution than on the bare GCE. A shift of the potential to the lower energy values as well as the higher reduction current reflect the electrocatalytic effect of the MnTMPyP complex in the solution on the hydrogen peroxide electroreduction. Other conditions: 0.1 M PBS, pH 7.4, scan rate 0.05 V s^-1^.

**

**

**SUPPORTING FIGURE S5.** Electrochemical measurements with the GCE/MnTPyP macroelectrode (Peng et al. 2020) in the wheat plant extracts (dashed red lines) with additions of hydrogen peroxide (blue and green lines).

**References**

Peng R, Offenhäusser A, Ermolenko Y, Mourzina Y (2020) Biomimetic sensor based on Mn(III) meso-tetra(N-methyl-4-pyridyl) porphyrin for non-enzymatic electrocatalytic determination of hydrogen peroxide and as an electrochemical transducer in oxidase biosensor for analysis of biological media. Sensors and Actuators B: Chemical:128437 doi:https://doi.org/10.1016/j.snb.2020.128437
